# Risk Factors for Difficult Three-Port Laparoscopic Cholecystectomy

**DOI:** 10.7759/cureus.71680

**Published:** 2024-10-17

**Authors:** Goshi Fujimoto, Takashi Deguchi, Junya Shirai, Kentaro Saito

**Affiliations:** 1 Gastroenterological Surgery, Koga Community Hospital, Yaizu, JPN

**Keywords:** acute cholecystitis, laparoscopic cholecystectomy, minimally invasive surgery, reduced-port surgery, three-port laparoscopic cholecystectomy, upper abdominal surgery

## Abstract

Background

Standard laparoscopic cholecystectomy (LC) is a four-port technique in which a camera port and three additional ports are used. The advantages of minimally invasive surgery with reduced-port surgery have been reported. However, evidence on the indications for minimally invasive surgery in patients with severe acute cholecystitis or previous upper abdominal surgery in whom laparoscopic surgery is considered challenging is limited. Therefore, this study aimed to explore the factors that complicate reduced-port LC.

Methods

Data from 47 consecutive patients who underwent three-port LC using two 5 mm ports and 12 mm umbilical ports for symptomatic cholecystolithiasis, chronic cholecystitis, and acute cholecystitis between November 2021 and November 2023 by a single surgeon were retrospectively collected. Noncomplete LC was defined as a change of 5 mm to 12 mm port, the addition of ports, a change to subtotal cholecystectomy, or open conversion cholecystectomy. The patients were divided into two groups according to complete or noncomplete LC, and the risk factors that might have contributed to noncomplete LC were explored.

Results

Among the 47 patients, the median (range) age was 74 (25-97) years, 21 were men and 26 were women, 30 (63.8%) had acute cholecystitis, and 21 (44.7%) underwent emergency LC. No conversion to open cholecystectomy was performed.

Six of the 47 patients had noncomplete LC, three of whom were converted to subtotal cholecystectomy, one had the midepigastric port changed from 5 mm to 12 mm to use an automatic anastomosis device, and two were converted to subtotal cholecystectomy with a 12 mm midepigastric port. In the univariate analysis, the noncomplete LC group had significantly more cases of preoperative gallbladder drainage and a smaller body mass index than the complete group. No significant differences were found in previous epigastric surgeries or in the presence of acute cholecystitis. Postoperative outcomes showed a significantly longer operative time, more intraoperative blood loss, longer postoperative hospital stay, and higher Estimation of Physiologic Ability and Surgical Stress and surgical stress score in the noncomplete LC group than in the complete group.

Conclusions

Three-port LC may be difficult to perform in patients with preoperative gallbladder drainage and severe scarring of the gallbladder neck. For patients with risk factors for three-port LC, adequate manpower and early conversion to subtotal or open cholecystectomy are necessary to avoid intraoperative complications. Further studies are required to determine significant risk factors for noncomplete LC.

## Introduction

The efficacy of reduced-port surgery, including single-port laparoscopic cholecystectomy (LC; single-incision laparoscopic cholecystectomy (SILC)) [1-5], two-port LC [6-8], and three-port LC [9,10], has been reported. However, little evidence exists for minimally invasive surgery in patients with severe acute cholecystitis, previous abdominal surgery, or poor performance status (PS) [1,3,11,12]. Compared with conventional four-port LC, three-port LC has better cosmetic results and can be performed safely without increased complications [10]. Additionally, three-port LC can be performed by two surgeons (operating surgeon and videoscope holder) using the same device as four-port LC, which leads to cost benefit [13,14]. In this study, all patients requiring LC, including those with a history of emergency or abdominal surgery, underwent three-port LC using an umbilical camera port and two 5 mm ports to determine the factors that complicate three-port LC.

## Materials and methods

Study design and patients

Forty-seven consecutive patients who underwent three-port LC for symptomatic cholelithiasis, chronic cholecystitis, and acute cholecystitis at Koga Community Hospital, Yaizu, Shizuoka, Japan, between November 2021 and November 2023 were included in this study. All operations were performed by the same surgeon (G.F.), and data were retrospectively collected.

This study was approved by the Ethics Committee of Koga Community Hospital (approval no.: 2023-11) and registered in the University Hospital Medical Information Network Clinical Trial Registry (UMIN000055473). All data were subject to strict privacy policies. The requirement for informed consent from the patients was waived by the ethics committee owing to the retrospective nature of this study, which used anonymized data.

Surgical procedure and treatment strategy

**Figure 1:**
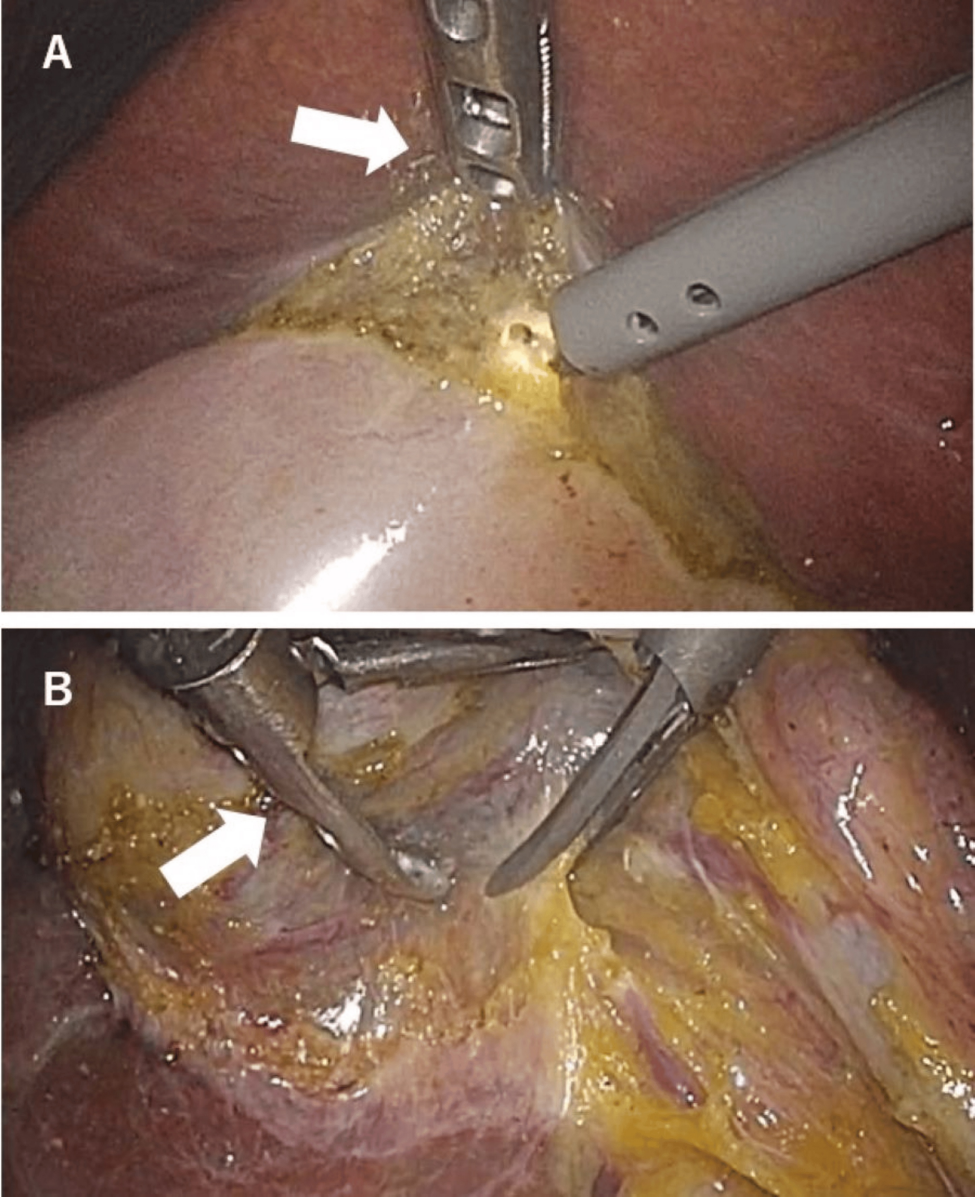
Gallbladder retraction method characteristic of three-port laparoscopic cholecystectomy (LC) (A) Traction of the incised gallbladder serosa and application of countertraction using the weight of the gallbladder. (B) Opening forceps to tract the gallbladder.

The three-port LC was started with the insertion of a 12 mm port for the video laparoscope at the umbilicus using open laparotomy (Hasson’s technique). The abdomen was insufflated with carbon dioxide to a pressure of approximately 10 mm Hg. The operating surgeon and videoscope holder stood on the patient’s left side and added a 5 mm port in the midepigastric and right subcostal regions. The patients were positioned with the head side up and right side up. A 5.4 mm diameter flexible camera was used. The surgeon operated via two 5 mm ports while the assistant manipulated the cameras. Techniques such as applying countertraction using the weight of the gallbladder or opening forceps to tract the gallbladder were employed (Figure 1). If abdominal wall adhesions interfered with surgery, the camera was inserted through the camera port or the 5 mm port to secure the operative field, and the adhesions were resected. A critical view of safety (CVS) was identified, and the cholecystic artery and duct were clipped and resected. If the CVS technique was difficult to perform, fundus-first cholecystectomy with bailout technique was performed. Intraoperative cholecystocentesis was performed to deflate the gallbladder when a distended gallbladder interfered with the surgery. The gallbladders were stored in bags (EZ Purse; Hakko Co., Ltd., Medical Device Division, Tokyo, Japan) and retrieved through the umbilical port. Large gallstones were placed in a bag, crushed intra-abdominally, and removed to avoid umbilical wound extension. Abdominal drains were not placed unless there was a high risk of postoperative bleeding or bile leakage. The group that completed the three-port LC using the above method was defined as the complete LC group. If the risk of liver or bile duct injury (BDI) was high, the patients underwent subtotal cholecystectomy or open surgery. Patients with severe scarring or intra-abdominal adhesions that made the laparoscopic approach or bleeding control difficult could be converted to open surgery. Noncomplete LC was defined as a change of 5 mm to 12 mm port, the addition of ports, a change to subtotal cholecystectomy, or open conversion cholecystectomy.

Data collection

All patients with symptomatic cholecystolithiasis, chronic cholecystitis, or acute cholecystitis who could tolerate the surgery were included in the three-port laparoscopic cholecystectomy. No age restrictions were required, and patients with a history of abdominal surgery, emergency surgery, or severe acute cholecystitis were also included. However, patients with preoperative suspicion of gallbladder cancer were excluded. Medical records were retrospectively reviewed, and patient information, including age, sex, body mass index (BMI), American Society of Anesthesiologists' physical status classification, preoperative Charlson Comorbidity Index score, preoperative antiplatelet or anticoagulant intake, preoperative or coexisting acute cholangitis or pancreatitis, severity of acute cholecystitis according to Tokyo Guidelines 2018 (TG18), preoperative gallbladder drainage, days from onset to surgery, size of the gallstone, coexisting fatty liver or cirrhosis, white blood cell count, C-reactive protein level, prognostic nutritional index (PNI) score, Surgical Apgar Score, Estimation of Physiologic Ability and Surgical Stress (E-PASS) score, and Physiological and Operative Severity Score for the Enumeration of Mortality and Morbidity (POSSUM) score, was collected. Surgical outcomes, including operative time, intraoperative blood loss, visual analog scale (VAS) score of postoperative day one pain, abdominal drain placement, postoperative complications, and duration of postoperative hospital stay, were recorded. Postoperative complications included all grades of the Clavien-Dindo classification [15]. The patients were divided into two groups according to complete or noncomplete LC, and the risk factors that might have contributed to noncomplete LC were explored.

Statistical analyses

Categorical variables are presented as numbers and percentages. They were compared between the groups using Fisher’s exact or χ^2^ test, as appropriate. Univariate analyses were performed to evaluate the clinical risk factors for noncomplete LC. All statistical inferences were assessed at a two-tailed significance level of 5%. Statistical significance was set at P < 0.05. All statistical analyses were performed using EZR (Saitama Medical Center, Jichi Medical University, Saitama, Japan), a graphical user interface for the R software version 3.5.1 (The R Foundation for Statistical Computing, Vienna, Austria) [16].

## Results

Of the 47 patients, the median (range) age was 74 (25-97) years; 21 (44.7%) were men, 26 (55.3%) were women, 30 (63.8%) had acute cholecystitis, and 21 (44.7%) underwent emergency LC. An information drain was placed in two patients: one owing to intraoperative bleeding exceeding 100 g and the other because the cystic duct was resected and sutured near the common bile duct. Both patients successfully underwent three-port LC. None of the patients indicated intraoperative cholangiography. No BDI or conversion to open cholecystectomy was performed.

Six of the 47 (12.8%) patients had noncomplete LC, three of which were converted to subtotal cholecystectomy, one had the midepigastric port changed from 5 mm to 12 mm, and two were converted to subtotal cholecystectomy with a 12 mm midepigastric port. No conversion to four-port LC was observed.

Risk factor for noncomplete three-port LC

Univariate analysis showed that BMI (complete vs. noncomplete LC: 23.4 vs. 18.4, *p* = 0.02) and preoperative gallbladder drainage (complete vs. noncomplete LC: 2.5% vs. 50%, *p* = 0.04) were significantly associated with noncomplete LC (Table 1).

**Table 1:** Univariate analysis to detect the risk factors for noncomplete laparoscopic cholecystectomy (LC) Statistical significance is set at P < 0.05. ^a^Data are presented as medians (ranges). ^b^Data are presented as medians (interquartile ranges). CCI: Charlson Comorbidity Index, ASA-PS: American Society of Anesthesiologists' physical status, TG18: Tokyo Guidelines 2018, PNI: prognostic nutritional index, E-PASS: Estimation of Physiologic Ability and Surgical Stress, POSSUM: Physiological and Operative Severity Score for the Enumeration of Mortality and Morbidity, WBC: white blood cells, CRP: C-reactive protein

	Complete LC (n = 41)	Noncomplete LC (n = 6)	P value
Age, years^a^	70 (25–97)	73 (53–85)	0.667
Sex, n (%)			0.386
Male	17 (41.4%)	4 (66.7%)	
Female	24 (58.5%)	2 (33.3%)	
BMI^a^	23.4 (13.5–34.7)	18.4 (12.5–25.3)	0.021^*^
CCI^a^	4 (1–9)	3 (0–5)	0.172
ASA-PS class, n (%)			0.416
1, 2	22 (53.7%)	2 (33.3%)	
3, 4	19 (46.3%)	4 (66.7%)	
Previous epigastric surgery, n (%)			1
+	2 (4.9%)	0 (0%)	
–	39 (95.1%)	6 (100%)	
Antiplatelet/anticoagulant, n (%)			0.395
+	13 (31.7%)	3 (50%)	
–	28 (68.3%)	3 (50%)	
PNI^b^	45.7 (39.1–51.1)	37.6 (31.2–41.9)	0.071
Surgical Apgar Score^b^	8 (7–8)	8 (6.25–9.75)	0.317
E-PASS PRS^b^	0.439 (0.31–0.789)	0.515 (0.421–0.567)	0.905
POSSUM physiological score^b^	19 (16–23)	23 (20–28.25)	0.062
POSSUM operative score^b^	7 (7–10)	7 (7–7)	0.303
Fatty liver, n (%)			1
+	4 (9.8%)	0 (0%)	
–	37 (90.2%)	6 (100%)	
Cirrhosis, n (%)			1
+	1 (2.4%)	0 (0%)	
–	40 (97.6%)	6 (100%)	
WBC, /μL^b^	6470 (4660–8550)	6780 (5078–10043)	0.211
CRP, mg/dL^b^	0.83 (0.09– 5.19)	1.8 (0.58–2.83)	0.757
Preoperative pancreatitis, n (%)			1
+	2 (4.9%)	0 (0%)	
–	39 (95.1%)	6 (100%)	
Preoperative cholangitis, n (%)			0.637
+	10 (24.4%)	2 (33.3%)	
–	31 (75.6%)	4 (66.7%)	
Acute cholecystitis, n (%)			0.396
+	25 (61.0%)	5 (83.3%)	
–	16 (39.0%)	1 (16.7%)	
TG18, n (%)			0.053
I or cholelithiasis, chronic cholecystitis	36 (87.8%)	3 (50%)	
II–III	5 (12.2%)	3 (50%)	
Gallstone size, mm^a^	5 (0–40)	4 (0–22)	0.932
Emergency surgery, n (%)			0.204
+	20 (48.8%)	1 (16.7%)	
–	21 (51.2%)	5 (83.3%)	
Preoperative gallbladder drainage, n (%)			0.039*
+	1 (2.4%)	40 (97.6%)	
–	2 (33.3%)	4 (66.7%)	
Duration from onset to surgery, days^b^	7 (2–25)	42.5 (14.75–56)	0.104

TG18 grades II-III, POSSUM physiological score, and PNI score < 40 tended to be associated with noncomplete LC, albeit without statistical significance. Previous epigastric surgery was not associated with noncomplete LC.

Outcomes of LC

Univariate analysis showed that longer operation time (complete LC vs. noncomplete LC: 59 vs. 82 min, *p* = 0.02), more intraoperative blood loss (complete LC vs. noncomplete LC: 11 vs. 33 g, *p* = 0.04), longer postoperative hospital stay (complete LC vs. noncomplete LC: 15 vs. 45 days, *p* = 0.020), and high E-PASS surgical stress score (SSS) (complete LC vs. noncomplete LC: -0.30 vs. -0.28, *p* = 0.017) were associated with the noncomplete LC group (Table 2). The VAS scores showed no significant differences between the two groups.

**Table 2:** Outcomes of laparoscopic cholecystectomy (LC) Statistical significance is set at p < 0.05. ^a^Data are presented as medians (interquartile ranges). E-PASS: Estimation of Physiologic Ability and Surgical Stress, SSS: surgical stress score, CRS: comprehensive risk score, VAS: visual analog scale

	Complete LC (n = 41)	Noncomplete LC (n = 6)	P value
Operative time, min^a^	59 (44–69)	81 (67.5–91.5)	0.0201^*^
Blood loss, g^a^	5 (2–10)	15 (10–43)	0.044^*^
Complications, n (%)			0.322
+	7 (17.1%)	2 (33.3%)	
–	34 (82.9%)	4 (66.7%)	
VAS^a^	1 (0–1)	0.5 (0–1)	0.5
Drain placement, n (%)			1
+	2 (4.9%)	0 (0%)	
–	39 (95.1%)	6 (100%)	
Duration of administration, days^a^	5 (3–9)	24 (7.5–87)	0.021^*^
E-PASS SSS^a^	–0.301 (–0.312 to –0.293)	–0.279 (–0.291 to –0.273)	0.017^*^
E-PASS CRS^a^	–0.206 (–0.338 to 0.121)	–0.119 (–0.89 to –0.0637)	0.949

## Discussion

The three-port and conventional four-port LCs can be performed safely; the midepigastric ports used in three-port LCs have been either 10 mm or 5 mm ports in previous studies. In a study of three-port LC using a 10 mm midepigastric port, the operative time, success rate, analgesic requirement, and postoperative hospital stay were similar between the three- and four-port LCs [10]. Another study showed that three-port LC was associated with a shorter postoperative hospital stay and less port site pain compared with four-port LC [17]. In a study of three-port LC using a 5 mm midepigastric port, the success rate, operative time, and VAS score of three- and four-port LCs were similar. In 2% of patients who underwent three-port LC, a fourth trocar (5 mm) was added to insert a retractor to clarify Calot’s triangle or the operative field. The number of analgesic injections was significantly smaller in the three-port LC group [18]. The three-port LC has a low-cost cosmetic advantage over the conventional four-port LC [18]. In the study excluding acute cholecystitis cases, two of the 315 (0.63%) patients who underwent three-port LC were converted to open cholecystectomy owing to anatomical abnormalities [12].

Reduced-port LC is safe for patients with acute cholecystitis [19]. In patients with cholecystitis who underwent three-port LC, some were converted to conventional four-port laparoscopic surgery because of inadequate visualization or to open surgery because of difficulty in dissection [2]. Although previous studies that performed three-port LC for acute cholecystitis excluded patients with a history of upper abdominal surgery [2,9], this study is important because it included patients with a history of upper abdominal surgery. In a study of two-port LC (umbilical incision as in SILS and 5 mm midepigastric incision) including cholecystitis, 1.8% had an additional port and 1.6% were converted to open cholecystectomy. The main reasons for surgical conversion are ambiguous anatomy, severe primary inflammation, bleeding control, and severe adhesions owing to previous abdominal surgery [8]. SILS for acute cholecystitis requires an additional port to assist with dissection [4], and the incidence of moderate-to-mild acute cholecystitis is significantly greater in cases with open conversion [19].

Incisional pain was significantly less in the group with three 5 mm ports than in the group with two 10 mm ports and one 5 mm port [14]. Therefore, in this study, a 5 mm midepigastric port was used instead of a 10 mm port to reduce pain. Furthermore, a 5 mm port allows a 5 mm camera to be inserted and adhesions to the abdominal wall to be resected from a different view than the umbilical camera port. The first umbilical 12 mm port was inserted using open laparotomy (Hasson’s technique) as per the operator’s preference. No differences were observed between open and closed techniques for vascular or visceral injury. The open technique was reported to have a lower risk of failed entry than the closed technique [20]. In elective LC, the use of a retrieval bag does not improve surgical site infection, and not using a bag leads to smaller fascial incisions, reducing the risk of postoperative pain and port site hernia [21]. However, if the gallstone size exceeds the length of the umbilical incision, it can be crushed inside the bag and removed; therefore, we consider the bag useful. In this study, gallstones up to 40 mm in size were observed, and gallstones > 20 mm were observed in five out of 47 cases. In all cases, extension of the fascial incision was avoided. In addition, no port site hernias were observed.

Although drains were not usually placed in this study, a drain was placed in two cases. One patient underwent emergency surgery with coagulopathy for grade II acute cholecystitis, and an information drain was placed to check for postoperative bleeding because intraoperative bleeding exceeded 100 g. The other patient had a 22 mm gallstone impacted in the neck of the gallbladder, which was retrieved via a small incision at the fundus of the gallbladder. Because the cystic duct was resected and sutured near the common bile duct, there was a high risk of bile leakage, and an information drain was placed. No postoperative bleeding or bile leakage was observed in either patient, and the drain was removed the day after surgery.

Comparing the five patients in the subtotal cholecystectomy group with the 42 patients in the total cholecystectomy group, significantly more cases of TG18 grade II-III (*p* = 0.029), and preoperative cholecystic drainage (*p* = 0.027) were performed in the subtotal cholecystectomy group. Comparing the three patients who had the midepigastric port changed to 12 mm with the 44 patients who required no change, the duration from onset to surgery was significantly longer in the former group than in the latter group (*p* = 0.033).

The reason for the significantly lower BMI in the noncomplete LC group in this study is unknown. The E-PASS SSS was calculated based on operative time, blood loss, and extent of skin incision. As no cases of laparotomy in this study meant no difference in the extent of skin incision, the significant difference in E-PASS SSS was owing to operative time and blood loss.

In summary, acute cholecystitis of TG18 grades II-III, preoperative gallbladder drainage, and a longer duration from onset to surgery were considered risk factors complicating three-port LC completion. This study is noteworthy compared to previous studies because it included patients with emergency surgeries, severe acute cholecystitis, a history of abdominal surgery, and poor PS. However, these factors were not found to be risk factors complicating three-port LC, except for severe acute cholecystitis. Patients with noncomplete LC tend to have a longer operative time and more blood loss; therefore, the operative schedule should be effectively planned in advance, and if anemia is detected preoperatively, a blood transfusion should be prepared.

The present study has some limitations. First, this was a single-center, retrospective, observational study with a relatively small population. Further studies are required to determine significant risk factors for noncomplete LC. Second, three-port LC in this study was performed by a single surgeon. The difficulty level of LC may differ among surgeons. Third, although this study included port conversion and conversion to subtotal cholecystectomy in the definition of noncomplete LC in addition to port addition and open conversion established in previous studies, other risk factors remain to be detected.

## Conclusions

Of the consecutive 47 patients who underwent three-port LC using a 12 mm umbilical camera port and two 5 mm ports, six could not undergo successful completion. The risk factors for difficult three-port LC are moderate-to-severe acute cholecystitis, preoperative gallbladder drainage, and a longer duration from onset to surgery. In cases without risk factors, three-port LC can be completed more reliably, thus contributing to a reduction in manpower and medical costs than conventional four-port LC. This study included patients with emergency surgery cases, severe acute cholecystitis, a history of abdominal surgery, and poor PS. However, these factors were not revealed to be risk factors for difficult three-port LC, except for severe acute cholecystitis. Patients with noncomplete LC tend to have a longer operative time and greater blood loss than those with complete LC; therefore, preparations, including scheduling, blood transfusions, and manpower, are important.
